# Towards the prevention of acute lung injury: a population based cohort study protocol

**DOI:** 10.1186/1471-227X-10-8

**Published:** 2010-04-27

**Authors:** Sweta J Thakur, Cesar A Trillo-Alvarez, Michael M Malinchoc, Rahul Kashyap, Lokendra Thakur, Adil Ahmed, Martin K Reriani, Rodrigo Cartin-Ceba, Jeff A Sloan, Ognjen Gajic

**Affiliations:** 1 Multidisciplinary Epidemiology and Translational Research in Intensive Care (M.E.T.R.I.C.), Division of Pulmonary and Critical Care Medicine, Mayo Clinic College of Medicine, Rochester, Minnesota, USA

## Abstract

**Background:**

Acute lung injury (ALI) is an example of a critical care syndrome with limited treatment options once the condition is fully established. Despite improved understanding of pathophysiology of ALI, the clinical impact has been limited to improvements in supportive treatment. On the other hand, little has been done on the prevention of ALI. Olmsted County, MN, geographically isolated from other urban areas offers the opportunity to study clinical pathogenesis of ALI in a search for potential prevention targets.

**Methods/Design:**

In this population-based observational cohort study, the investigators identify patients at high risk of ALI using the prediction model applied within the first six hours of hospital admission. Using a validated system-wide electronic surveillance, Olmsted County patients at risk are followed until ALI, death or hospital discharge. Detailed in-hospital (second hit) exposures and meaningful short and long term outcomes (quality-adjusted survival) are compared between ALI cases and high risk controls matched by age, gender and probability of developing ALI. Time sensitive biospecimens are collected for collaborative research studies. Nested case control comparison of 500 patients who developed ALI with 500 matched controls will provide an adequate power to determine significant differences in common hospital exposures and outcomes between the two groups.

**Discussion:**

This population-based observational cohort study will identify patients at high risk early in the course of disease, the burden of ALI in the community, and the potential targets for future prevention trials.

## Background

**Figure 1:**
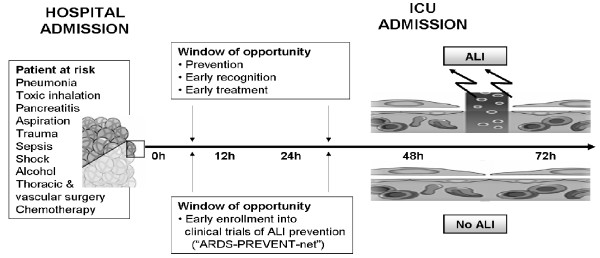
**Proposed "two hit" model of ALI development: the window of opportunity exists for the potential ALI prevention strategies**.

Acute lung injury (ALI) and its more severe form Acute respiratory distress syndrome (ARDS) are common and devastating complications after acute illness or injury with high morbidity and mortality, long term decrease in quality of life, and enormous costs related to intensive care and rehabilitation [1]. ALI is an example of a critical care syndrome with limited treatment options once the condition is fully established. Despite improved understanding of the pathophysiology of ALI, the clinical impact has been limited to improvements in supportive treatment [2,3]. Surprisingly little research has been done on the prevention of ALI. Preclinical studies support a "two hit" model of development of ALI whereby different exposures modify the expression of ALI in susceptible host [4]. Preliminary data suggest that ALI is rarely present at the time of hospital admission but develops over a period of hours to days in subsets of patients with predisposing conditions such as pneumonia, sepsis, trauma, shock and corresponding medical and surgical interventions [5-12]. To this extent, ALI may be viewed as potentially preventable hospital complication similar to stress ulcer bleeding, venous thromboembolism or nosocomial infections. Previous clinical studies enrolled patients after ICU admission, often with already established ALI, beyond the window of meaningful mechanistic studies and potential prevention strategies [13-15]. Not surprisingly, many treatments targeting the mechanisms identified in preclinical studies have failed to improve patient outcomes despite compelling preclinical data [16-19]. It is likely that, inadequate and delayed recognition of patients at risk and the subsequent development of the full blown syndrome have obscured the therapeutic window. ALI usually develops during the first hours of ICU admission, and often is the very reason for ICU admission. On the other hand, the majority of patients with predisposing conditions never develop ALI and they may never been admitted to the ICU making the enrollment of unselected patients into ALI prevention studies neither feasible nor efficient [12,10]. A meaningful approach to ALI prevention therefore ought to be based on identifying patients at risk earlier than what is currently done (at the time of hospital admission, rather than ICU admission, Figure 1) [10].

## Methods/Design

**Figure 2:**
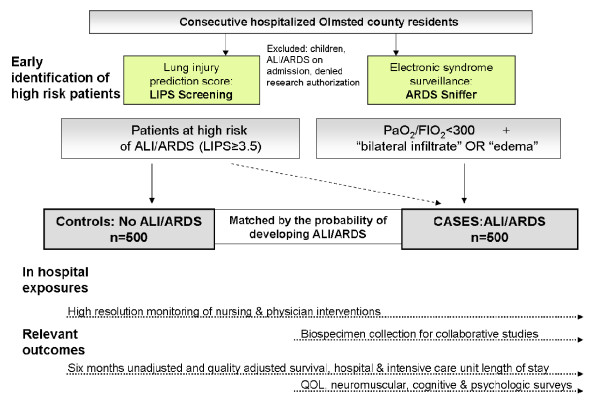
**Outline of the study design**.

In this population based cohort study the investigators will identify patients at risk early in the course of the disease and *before *the development of ALI (at the time of hospital admission). Detailed in-hospital exposures, short and long term outcomes will be compared between patients at high risk who do and do not develop ALI. (Figure 2)

### Inclusion Criteria

Olmsted County residents more than 18 years of age admitted to the two Mayo Clinic Rochester hospitals with one or more of the ALI predisposing conditions (sepsis, pneumonia, aspiration, pancreatitis, shock, high risk trauma, and high risk surgery). (Table 1) Exclusion criteria are listed in Table 2. Institutional review board has approved the study protocol.

**Table 1:** Predisposing conditions and modifiers available before or within six hours after hospital admission used to calculate the lung injury prediction score (LIPS):[22].

*Risk factors*	Measurement	Definition
Pneumonia	Yes/No	Consensus Conference[24]
Sepsis	Yes/No, severe	SCCM-ACCP definition[25]
Pancreatitis	Yes/No	Practice guidelines in acute pancreatitis[26]
Aspiration (pre-admission)	Yes/No	Inhalation of food or gastric contents[27]
High risk trauma	Lung contusion, smoke inhalation, near-drowning, multiple bone, brain injury	From Derdak [28]
High risk surgery	Aortic vascular, spine, thoracic, acute abdomen, emergency	From Arozullah et al [29,30]
*Risk Modifiers*		
Alcohol use	Yes/No, amount (# of drinks a week)	More than 2 drinks per day or a history of alcohol-related illness or admission[31,32]
Smoking	Never/former/current/# of pack-years	Substance Abuse and Mental Health
Diabetes mellitus*	Yes/No	Diabetes care 2009[33]
Interstitial lung disease	Yes/No	ATS-ERS Consensus classification of IIP[34]
Chemotherapy	Yes/No	Custom, cancer chemotherapeutic drugs during the 6 months prior to hospitalization
Tachypnea (respiratory rate>30),		Based on the worst value during the first 6 hours
High inspired oxygen concentration (FIO2>0.35)		Based on the worst value during the first 6 hours
Hypoalbuminemia		Serum albumin <3.5 g/dL, the absence of measurement considered normal

*Only if sepsis

**Table 2:** Exclusion criteria.

Exclusion Criteria	Justification
Acute lung injury or pulmonary edema already present at the time of hospital admission	Unable to assess for development of outcome of interest
Admitted for comfort or hospice care only	Missing predictor and outcome variables
Denied the use of medical records for research (~5%)	Self explanatory
Patients admitted for cardiac telemetry, coronary care unit, low risk elective surgeries, labor and delivery	Very low risk of outcome of interest
Children	Different risk factors and outcome
Hospital readmission	Complexity of analysis
Hospital transfer	Exposure to health care interventions

### System-wide electronic surveillance

Electronic medical records (EMR) facilitate early recognition of specific study criteria using Boolean combinations of clinical variables and natural language processing. In this study we are planning to use a customized, integrative relational research database that contains a near-real time (15 minutes delay) copy of electronic medical records (ICU DataMart). To identify Olmsted County residents at risk admitted to the two Mayo Clinic hospitals, ICU DataMart uses 9-digit ZIP code and a specific nursing unit codes and generates e-mail and/or pager alert within 15 minutes from the time the patient is assigned a bed in the receiving unit. Patients admitted for labor and delivery, specific procedures (cardiac catheterization), cardiac telemetry, coronary care unit, low risk elective surgeries and children are excluded from the alerts. Screening for ALI development is performed by previously validated ARDS "sniffer" [20,21]. The electronic alert is triggered by the following combination of observations: 1) qualifying arterial blood gas analysis: the ratio of partial pressure of oxygen to inspired oxygen concentration (PaO_2_/FIO_2_) <300 and 2) qualifying chest radiograph report: free text Boolean query containing trigger words: ("bilateral" AND "infiltrate") OR "edema" The ARDS sniffer demonstrated excellent negative predictive value (0.99, 95% CI 0.98 to 1.00)[20]. ARDS-sniffer has been continuously running since December of 2005 allowing for prospective identification of ALI cases. Access to the database is accomplished through password protected open database connectivity (ODBC) using JMP and SAS statistical software (SAS Institute Inc., Cary, NC, USA).

### Early identification of patients at risk of ALI (lung injury prediction score - LIPS)

To facilitate enrollment of patients into mechanistic and outcome studies as well as future ALI prevention trials, we have recently developed an ALI prediction model (Lung Injury Prediction Score: LIPS, Table 1)[22]. LIPS incorporates demographic, and clinical characteristics at the time of, and before, hospital admission. Risk factors for ALI that were identified in at least two previous studies were used in model development. LIPS points were determined based on parameter estimates from the logistic regression model, taking into consideration results from our previous studies. The model accurately discriminated between the patients who did and did not develop ALI with an area under the receiver operating curve of 0.85 (95% CI 0.80 to 0.89) [22].

Twice daily (7 AM and 5 PM, Monday-Saturday) trained study coordinators review syndrome surveillance alerts of new Olmsted County admissions and apply LIPS points to patients who fulfill the inclusion criteria according to LIPS score sheet. ODBC MS Access database tool is used for the collection of individual patient data in a systematic way. The database automatically links MS Access to ICU Datamart server and imports new patients from ICU Datamart to the LIPS database. The database also links automatically to the IRB research authorization web site identifying the patients that have approved the use of their medical data for research.

### Validation of the primary outcome (ALI)

Primary outcome is the development of ALI at any time during the hospital stay. Trained investigators review each ARDS sniffer alert (see above) and confirm the presence or absence of ALI according to the standard definition based on the American-European consensus conference [21]. The absence of left atrial hypertension as the principal explanation for pulmonary edema is confirmed by integrated clinical evaluation based on the following:

• Echocardiography (E/E'<15, EF>45)

• Pulmonary artery occlusion pressure (PAOP) ≤ 18 cm H2O

• Central venous pressure (CVP < 15) cm H2O (higher cutoff in pulmonary hypertension)

• History of congestive heart failure/cardiogenic pulmonary edema

• Brain natriuretic peptide (BNP) <250 pg/mL (higher cutoff in renal failure)

• Response to preload reduction: brisk (hours) response to diuretics and/or positive pressure ventilation favors hydrostatic edema

This process yielded good interobserver agreement for differentiation between ALI and hydrostatic edema (Kappa value 0.83 in the most recent retrospective Olmsted county study)[10].

### Identification of in-hospital exposures

To identify candidate interventions for ALI prevention, we are planning to identify hospital acquired (2^nd ^hit) environmental exposures that may modify the risk of ALI and its consequences in high risk subgroups of patients residing in the Olmsted County community identified by the LIPS model. Timing and intensity of environmental exposures (Table 3) will be determined by the review of monitoring logs, nursing and physician interventions in the EMR by trained study coordinators blinded to ALI status. Only the exposures occurring before development of the ALI in cases and during the corresponding period of time in controls will be analyzed as modifiers of ALI development.

**Table 3:** Hospital (second hit) exposures that may modify the development of ALI in high risk patients.

	Variable	Measurements
**A. Exposures associated with development of ALI in preliminary studies**		
Treatment of infection	Antibiotics	Time to administration of adequate antibiotics
	Source control	List source, time to source control
Shock resuscitation	Early goal directed resuscitation	Time to completion of goal directed resuscitation
Blood transfusion	Red blood cells, fresh frozen plasma, platelets	Number of units, donor gender and pregnancies, storage age of red cells and platelets
	
Respiratory support	Inspired oxygen concentration (FIO_2_)	AUC, mean, median and maximum value during the exposure time of interest
	
	Ventilator tidal volumes (mL/kg predicted weight)	AUC, mean, median and maximum value during the exposure time of interest
	Peak and plateau airway pressures (cm H_2_O)	AUC, mean, median and maximum value during the exposure time of interest
	PEEP (cm H_2_O)	5-10 (reference), <5, >10 cm H_2_O
	
	Respiratory rate	AUC, mean, median and maximum value during the exposure time of interest
Medications	Amiodarone	Cumulative dose during the exposure time of interest
	Chemotherapy	Cumulative dose during the exposure time of interest
Other complications	Aspiration (in hospital)	Inhalation of food or gastric contents
	Medical or surgical misadventures	List complication (ICD-9 code E870-E879.9)
**B. Additional biologically plausible hospital exposures - exploratory analysis**		

**Mechanism**	**Variable**	**Measurements**
Inflammation	Corticosteroids, inhaled and systemic	Cumulative dose during the exposure time
	Statins	Cumulative dose during the exposure time
	Insulin	Cumulative dose during the exposure time
	ACE inhibitor	Cumulative dose during the exposure time
Oxidative stress	Antioxidants: *N*-Acetylcysteine, vitamin C, Vit E, Ω 3 fatty acids	Cumulative dose during the exposure time
Coagulation	Anticoagulants (heparin, coumadin)	Cumulative dose during the exposure time
	Antiplatelet agents (aspirin, clopidogrel)	Cumulative dose during the exposure time
Aspiration	Head of the bed position	Above horizontal
	Gastric tube feeding	Cumulative dose during the exposure time
	Sedative agents	Cumulative dose during the exposure time
	Narcotics	Cumulative dose during the exposure time
	Antipsychotics	Cumulative dose during the exposure time
	Gastric acid agents (H_2 _blockers, PPIs)	Cumulative dose during the exposure time
Metabolic	IV fluid (saline, Ringer, albumin)	Cumulative dose during the exposure time
	Furosemide	Cumulative dose during the exposure time
	Beta agonists, inhaled and systemic	Cumulative dose during the exposure time
	Laboratory findings: albumin, pH, glucose, osmolarity	The worst value during the exposure time of interest

AUC - area under the curve, PEEP - positive end expiratory pressure

PPI - proton pump inhibitor

### Bio specimen collection and storage for collaborative genomic and biomarker studies

Having identified both a robust phenotype and a detailed account of potentially important environmental exposures we will collect time sensitive peripheral blood samples for collaborative genome-wide association and plasma biomarker studies. Waste blood samples collected for routine clinical care will be collected at baseline (hospital admission), after 24 and 48 hours, and on the day of development of ALI (if outside these 3 time points).

### Attributable burden of ALI

To prioritize future ALI prevention strategies, we are planning to determine the attributable burden of ALI in the Olmsted County community by quantifying patient-centered outcomes attributable to this condition. The essential patient-centered outcomes (unadjusted and quality-adjusted survival, neurocognitive, neuropsychologic and neuromuscular complications, functional outcome, and quality of life) will be compared between patients who do and do not develop ALI. The instruments we have chosen to evaluate our patients are listed in Table 4. These instruments were selected to assess the key domains of patient centered outcomes without jeopardizing the ability and willingness of the patients to provide data. Specifically these instruments will give a general measure of Quality of life (QOL) (SF12), along with measures of physical, cognitive and psychological functioning and would provide a comprehensive picture of our patients' experience. Baseline (premorbid) functional status and QOL will be determined by in-hospital retrospective survey of the patients or their surrogates. After obtaining informed consent trained study coordinators establish if the patient is competent to complete the entire questionnaire by administering the mini mental test. If patient is deemed incompetent or too ill to complete the survey a surrogate will be identified to help fill the questionnaires. Follow-up contact information will be obtained and the patients or their surrogate who successfully complete the baseline survey will be contacted by telephone six month after index hospitalization.

**Table 4:** Patient-reported outcome assessment.

Variable	Instrument	Time(s) of assessment
Quality of life	SF-12 health survey	1. In hospital retrospective assessment of pre morbid QOL 2. 6 month follow up
Physical function	Barthel Index	1. In hospital retrospective assessment of pre morbid QOL 2. 6 month follow up
Neuromuscular complications	Overall neuropathy limitations scale	1. In hospital retrospective assessment of pre morbid QOL 2. 6 month follow up
Neurocognitive complications	Folstein mini-mental status scale	1. In hospital retrospective assessment of pre morbid QOL 2. 6 month follow up
Neuropsychological complications	1. Post-Traumatic Stress Syndrome (PTSS-10) (only at 6 months) 2. Yale depression scale	1. In hospital retrospective assessment of pre morbid QOL 2. 6 month follow up

### Strategies for subject retention

We selected survey instruments to minimize respondent burden. In preliminary testing, respondents completed the battery of tests in less than 15 minutes. At baseline the research assistant administer the questionnaire with the use of visual aids to help with answering the questions. When obtaining the consent, patients will be informed of the follow up survey and the same research assistant will contact the patients at six months. This will help in establishing a rapport and increasing the response rate at follow-up. The average retention rate for the surveys conducted by the QOL research team at our institution is in excess of 80%.

### Quality control and data management

LIPS study collects a large number of data regarding patient exposures and outcomes. To reduce measurement error, a comprehensive data quality assurance program will be employed. Data will be maintained on a secure server with nightly back up. Single Mayo logon LAN ID (local area network identification) provides access to the data to authorized individuals. Electronic range checks and validation rules will eliminate erroneous data entry and artifacts in numeric values. As an independent quality control measure periodic audits of the database will be performed. Routine descriptive statistics will be obtained at regular intervals to assure completeness of data collection.

### Statistical Analysis

An estimate for the probability of ALI development will be calculated for each patient based on the LIPS model. Because of subjective nature of clinical definitions used to identify the outcome of our study, it is necessary to assess interobserver variability. Interobserver agreement for ALI development will be assessed by calculating the Kappa statistic, using a threshold of 0.6 as an indication of acceptable interobserver agreement.

The calibration and discrimination of model will be assessed using the Hosmer-Lemeshow goodness-of-fit test and the receiver operating characteristic curve, respectively. An area under the ROC curve greater than 80% will be considered to be the evidence of good model discrimination. Using a LIPS cutpoint of 3.5 or more identified in the preliminary data, the comparison of the model prediction versus the actual event observed will be summarized by a simple 2 by 2 table reporting ALI propensity versus ALI development. Summary measures, based on the 2 by 2 table, will be reported: sensitivity, specificity, positive predictive value, negative predictive value, positive and negative likelihood ratios along with their 95% confidence intervals.

In a matched case-control design the associations suggested by preliminary clinical studies (Table 3) will be compared between ALI cases and high risk controls propensity matched by age, gender and the probability of ALI development at the time of hospital admission (LIPS, see above). Subpopulation analyses will be performed to determine what hospital exposures are associated with ALI in low/moderate ALI risk patients and what hospital exposures factors are associated with no ALI development in high ALI risk patients. The primary statistical endpoint is the development of ALI at any time during hospital stay. The matching is based on ALI risk at admission and the exposure period at risk. For example, if a hospitalized patient, who has an estimated propensity for developing ALI of 20% actually develops ALI 10 hours after hospital admission the exposures in matched control patient is only measured during the initial 10 hours after hospital admission. Paired statistics will be used for group comparisons. A conditional logistic regression model will be built in the case of baseline imbalances.

The statistical endpoints for determining attributable burden of ALI development in patients at risk are unadjusted and quality adjusted survival after hospital admission. All patients will be followed until death or study conclusion, and patients who survive will be censored at the last date known to be alive. In addition, we will assess which hospital exposures, impact the long-term survival and quality-adjusted survival in both ALI patients and high risk controls. We will use Kaplan-Mayer survival curves to depict survival differences in these subgroups. In order to understand the impact of ALI on quality of life, we will compare the l quality of life measures between the ALI patients and controls, taking into account the correlations between serial (baseline and follow up) QOL measurements. We will carry out quality-adjusted life years (QALY) analyses to incorporate the QOL-related health state utility variables into the survival analysis. We will combine survival and quality of life using the time spent in specific "health state" (ventilator, ICU, hospital, nursing home, home) to describe the quality adjusted survival of ALI patients according to the following formula:

We expect that missing data will be minimized due to the error checking capability of our data entry system. We will handle missing data in a number of ways including complete case analysis and imputation via nearest neighbor, mean value, last value, and zero value carried forward approaches. Multiple approaches are used so that the sensitivity of results to alteration in imputational assumptions may be assessed.

### Sample size considerations

Planned enrollment of 500 ALI cases guarantee an adequate sample size for LIPS validation. If we have 500 ALI cases and 80% score above the threshold for the ALI model (sensitivity) then our precision will be .02 and the 95% CI would 0.78 to 0.82.

**Figure 3:**
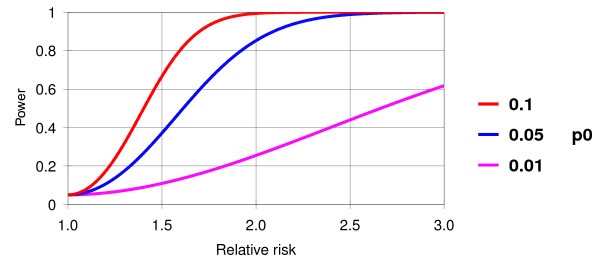
**Power for a matched case control study of 500 ALI patients matched to 500 controls where the risk factor occurs at 10%, 5%, or 1% prevalence in the controls and the false positive rate is 0.05**. As depicted, if 5% of the controls experience delayed fluid resuscitation which increases the risk of ALI by 2 fold, then the study has about 82% power to reject the null hypothesis.

A comparison of 500 ALI cases with 500 propensity matched high risk controls will allow us to determine moderately high associations (odds ratio >2.0) between common in-hospital exposures (prevalence >5%, i.e. variability in fluid management, transfusion, antibiotics, FIO2, mechanical ventilation) and ALI development (Figure 3). We will be able to identify only strong associations for less common in-hospital exposures (<5%).

Assuming a median survival of around 2 years in control population and ALI increases the risk of death by about 1.25, then the study has 80% power to reject the null hypothesis of equal survival of controls and ALI patients. A target of 100 ALI survivors and 100 controls assuming a standard deviation of the health related quality of life score among ICU patients of 10 units, the study has 80% power to detect a difference in self reported quality of life of 4.0 units or greater.

## Discussion

The LIPS study is a population based observational cohort study that aims to identify patients at high risk of developing ALI early in the course of illness (at the time of hospital admission), and compare the in-hospital (second hit) exposures and outcomes of patients at high risk who do and do not develop ALI. Analysis and comparison of exposures and outcomes between patients at high risk identified before ALI development is essential to understand clinical pathogenesis of ALI and design effective prevention strategies. By determining not only candidate interventions, but also the attributable burden of ALI, it will allow the prioritization of preventive strategies and future clinical trials.

Olmsted County offers a unique opportunity to study potential ALI prevention targets in a geographically defined population because all the critically ill patients from the county are admitted to Mayo Clinic hospitals. The use of population based sample eliminates the referral bias common in clinical studies performed at tertiary care institutions[23]. As a part of study design we have developed a near real time access to pertinent data in electronic medical records to identify patients from the community with or at high risk of ALI. The electronic infrastructure will greatly facilitate to conduct this population based study by minimizing study coordinator time necessary to screen large number of patients. Time-sensitive biospecimen collection in a population of patients with strictly defined phenotype and detailed capture of environmental exposures will allow for the development of important biospecimen repository for future collaborations in Genome Wide Association and plasma biomarker studies.

The principal limitation of our study is imposed by the broad nature and definition of the primary outcome of interest, ALI [21]. The exclusion of left atrial hypertension poses a particular challenge in the clinical assessment of ALI. In addition to using a standardized definition and extensive training of study personnel, all ALI cases will be reviewed by trained expert investigators. There is a possibility of ascertainment bias as we will depend on the clinicians caring for the patient to order the chest x-ray and the arterial blood gas necessary to diagnose ALI. Fortunately, almost all patients requiring respiratory support do have these tests performed routinely every day. It is therefore extremely unlikely that a significant ALI would occur without the need to perform the chest x-ray and the arterial blood gas as a part of clinical care.

Additional limitations of our approach come from the observational design of this study. First, because participants are not randomly assigned to the exposures under investigation, our study is particularly prone to indication bias, in that patients receiving certain therapies may be systematically different from those not receiving the therapies. If these differences are associated with the outcome of interest, the study results may be biased. Large sample size and a comprehensive collection of exposure variables will mitigate the potential bias by enabling our statisticians to adjust for any measured factor found to predict the use of each exposure under investigation. However, some important factors may be unknown or unmeasured, resulting in residual confounding and bias. The population based sample is clearly a strong point of our study. However, all patients will be treated in the two hospitals of the single teaching medical center, and, although internal validity will be high, the study results may not generalize to patients in other settings. Moreover, we will not be able to take advantage of additional variability in practice such as would be possible in multicenter studies involving different parts of the world. Long study period raises another question about whether the exposures, prognosis, and incidence of ALI will be stable enough to allow the proposed investigation. Fast pace changes in health care delivery and quality improvement initiatives could plausibly lead to the change in frequency of some of the proposed in-hospital exposures. Should changes in practice occur during the study period, our detailed observation of both practice and outcomes will give us an opportunity to correlate changes in practice with the development and outcome of ALI and possibly be able to make stronger causal inferences from the observed associations.

This causal translational research study will not affect the outcome of studied patients (this is a non-intervention epidemiologic study) but will help in better understanding the clinical pathogenesis of ALI and the design of future ALI prevention strategies. Since the therapeutic options are limited once ALI develops, the prevention is paramount. Unfortunately, effective prevention interventions do not currently exist, and our knowledge about clinical pathogenesis of ALI is limited. By identifying patients at high risk earlier (in the emergency department and operating room), and collecting biospecimens and clinical data before ICU admission we hope to improve our understanding of ALI and identify targets for future quality improvement interventions and ALI prevention trials. Previous studies have concentrated on patients in the ICU with already established ALI. Hence the inferences from these studies with regards to ALI pathogenesis and potential prevention targets are limited.

Potential future prevention strategies include, but are not limited to 1) quality improvement interventions to limit specific hospital acquired exposures (delayed treatment of infection and shock, aspiration triggers, high tidal volume ventilation, plasma transfusion from alloimmunized donors), and 2) the use of systemic and inhaled anticoagulants, antiplatelet agents, anti-inflammatory drugs and antioxidants. Some of these therapies have already been tested in preliminary clinical trials with encouraging result. This is in contrast to uniformly negative results of mechanistic interventions when applied later in the course of illness, once ALI is established.

## Conclusion

This population based observational cohort study will define 1) the population of patients at high risk for ALI at the time of hospital admission 2) the most significant second hit in-hospital exposures that may modify the development and progression of ALI and 3) attributable burden of ALI in the community. The results will inform future mechanistic studies and clinical trials with an ultimate goal of preventing this devastating complication of critical illness.

## Competing interests

The authors declare that they have no competing interests.

## Authors' contributions

All authors made substantial contribution to the study design and methods. SJT, LT, AA and MKR drafted the manuscript and all other authors critically revised it for important intellectual content. MMM, JAS and OG performed the statistical analysis. OG, RC and CT conceived the study, and participated in its design and coordination. All authors read and approved the final manuscript.

## Pre-publication history

The pre-publication history for this paper can be accessed here:

http://www.biomedcentral.com/1471-227X/10/8/prepub

## References

[B1] Avecillas J Freire A Arroliga A Clinical epidemiology of acute lung injury and acute respiratory distress syndrome: incidence, diagnosis, and outcomes Clin Chest Med 2006 27 4 549 557 10.1016/j.ccm.2006.06.001 17085244

[B2] Wiedemann HP Wheeler AP Bernard GR Thompson BT Hayden D DeBoisblanc B Connors AF Jr Hite RD Harabin AL Comparison of two fluid-management strategies in acute lung injury New England Journal of Medicine 2006 354 2564 2575 10.1056/NEJMoa062200 16714767

[B3] Ventilation with lower tidal volumes as compared with traditional tidal volumes for acute lung injury and the acute respiratory distress syndrome. The Acute Respiratory Distress Syndrome Network N Engl J Med 2000 342 18 1301 1308 10.1056/NEJM200005043421801 10793162

[B4] Matthay MA Zimmerman GA Esmon C Bhattacharya J Coller B Doerschuk CM Floros J Gimbrone MA Jr Hoffman E Hubmayr RD Future research directions in acute lung injury: summary of a National Heart, Lung, and Blood Institute working group Am J Respir Crit Care Med 2003 167 7 1027 1035 10.1164/rccm.200208-966WS 12663342

[B5] Gajic O Dara S Mendez J Adesanya A Festic E Caples S Rana R St Sauver J Lymp J Afessa B Ventilator-associated lung injury in patients without acute lung injury at the onset of mechanical ventilation Crit Care Med 2004 32 9 1817 1824 10.1097/01.CCM.0000133019.52531.30 15343007

[B6] Rana R Fernandez-Perez ER Khan SA Rana S Winters JL Lesnick TG Moore SB Gajic O Transfusion-related acute lung injury and pulmonary edema in critically ill patients: a retrospective study Transfusion 2006 46 9 1478 1483 10.1111/j.1537-2995.2006.00930.x 16965572

[B7] Khan H Belsher J Yilmaz M Afessa B Winters JL Moore SB Hubmayr RD Gajic O Fresh-Frozen Plasma and Platelet Transfusions Are Associated With Development of Acute Lung Injury in Critically Ill Medical Patients Chest 2007 131 5 1308 1314 10.1378/chest.06-3048 17400669

[B8] Gajic O Rana R Winters JL Yilmaz M Mendez JL Rickman OB O'Byrne MM Evenson LK Malinchoc M DeGoey SR Transfusion-related acute lung injury in the critically ill: prospective nested case-control study Am J Respir Crit Care Med 2007 176 9 886 891 10.1164/rccm.200702-271OC 17626910 PMC2048675

[B9] Fernandez-Perez ER Sprung J Afessa B Warner DO Vachon CM Schroeder DR Brown DR Hubmayr RD Gajic O Intraoperative ventilator settings and acute lung injury after elective surgery: a nested case control study Thorax 2009 64 2 121 127 10.1136/thx.2008.102228 18988659

[B10] Shari G Cartin-Ceba R Trillo Alvarez C Li G Kashyap R Kojicic M Dong Y Poulose JT Herasevich V Cabello Garza JA Timing to the Onset of Acute Respiratory Distress Syndrome in a Population Based Sample Am J Respir Crit Care Med 2009 179 1_MeetingAbstracts A5099

[B11] Iscimen R Cartin-Ceba R Yilmaz M Khan H Hubmayr RD Afessa B Gajic O Risk factors for the development of acute lung injury in patients with septic shock: an observational cohort study Crit Care Med 2008 36 5 1518 1522 10.1097/CCM.0b013e31816fc2c0 18434908

[B12] Ferguson ND Frutos-Vivar F Esteban A Gordo F Honrubia T Penuelas O Algora A Garcia G Bustos A Rodriguez I Clinical risk conditions for acute lung injury in the intensive care unit and hospital ward: a prospective observational study Crit Care 2007 11 5 R96 10.1186/cc6113 17784960 PMC2556739

[B13] Hudson LD Milberg JA Anardi D Maunder RJ Clinical risks for development of the acute respiratory distress syndrome Am J Respir Crit Care Med 1995 151 2 Pt 1 293 301 7842182 10.1164/ajrccm.151.2.7842182

[B14] Gong MN Thompson BT Williams P Pothier L Boyce PD Christiani DC Clinical predictors of and mortality in acute respiratory distress syndrome: potential role of red cell transfusion Crit Care Med 2005 33 6 1191 1198 10.1097/01.CCM.0000165566.82925.14 15942330

[B15] Fowler AA Hamman RF Good JT Benson KN Baird M Eberle DJ Petty TL Hyers TM Adult respiratory distress syndrome: risk with common predispositions Ann Intern Med 1983 98 5 Pt 1 593 597 6846973 10.7326/0003-4819-98-5-593

[B16] Ketoconazole for early treatment of acute lung injury and acute respiratory distress syndrome: a randomized controlled trial. The ARDS Network Jama 2000 283 15 1995 2002 10.1001/jama.283.15.1995 10789668

[B17] Jepsen S Herlevsen P Knudsen P Antioxidant treatment with n-acetylcyesteine during adult respiratory distress syndrome: a prospective randomized placebo controlled study Crit Care Med 1992 20 819 923 10.1097/00003246-199207000-00004 1617983

[B18] Meade MO Jacka MJ Cook DJ Dodek P Griffith L Guyatt GH Survey of interventions for the prevention and treatment of acute respiratory distress syndrome Crit Care Med 2004 32 4 946 954 10.1097/01.CCM.0000120056.76356.AD 15071383

[B19] Wiedemann HP Arroliga AC Komara J Denver VA Welsh C Fulkerson WJ Jr MacIntyre N Mallatratt L Sebastian M Sladen R Randomized, placebo-controlled trial of lisofylline for early treatment of acute lung injury and acute respiratory distress syndrome Critical Care Medicine 2002 30 1 6 10.1097/00003246-200201000-00001 11902249

[B20] Herasevich V Yilmaz M Khan H Hubmayr RD Gajic O Validation of an electronic surveillance system for acute lung injury Intensive Care Med 2009 35 6 1018 1023 10.1007/s00134-009-1460-1 19280175 PMC2730460

[B21] Bernard GR Artigas A Brigham KL Carlet J Falke K Hudson L Lamy M Legall JR Morris A Spragg R The American-European Consensus Conference on ARDS. Definitions, mechanisms, relevant outcomes, and clinical trial coordination Am J Respir Crit Care Med 1994 149 3 Pt 1 818 824 7509706 10.1164/ajrccm.149.3.7509706

[B22] Cartin-Ceba R Trillo Alvarez C Li G Kashyap R Kojicic M Dong Y Poulose JT Herasevich V Cabello Garza JA Shari G Derivation of a Lung Injury Prediction Score (LIPS) To Identify Patients at High Risk of ARDS at the Time of Hospital Admission Am J Respir Crit Care Med 2009 179 1_MeetingAbstracts A4653

[B23] Seferian E Afessa B Gajic O Peters SG Hubmayr RD Keegan MT Is there referral bias in the critically ill? Crit Care Med 2006 43 12 Suppl A19 10.1097/00003246-200612002-00076 18828201

[B24] Calandra T Cohen J The international sepsis forum consensus conference on definitions of infection in the intensive care unit Crit Care Med 2005 33 7 1538 1548 10.1097/01.CCM.0000168253.91200.83 16003060

[B25] American College of Chest Physicians/Society of Critical Care Medicine Consensus Conference: definitions for sepsis and organ failure and guidelines for the use of innovative therapies in sepsis Crit Care Med 1992 20 6 864 874 1597042

[B26] Banks PA Practice guidelines in acute pancreatitis Am J Gastroenterol 1997 92 3 377 386 9068455

[B27] Marik PE Aspiration pneumonitis and aspiration pneumonia N Engl J Med 2001 344 9 665 671 10.1056/NEJM200103013440908 11228282

[B28] Derdak S Acute respiratory distress syndrome in trauma patients J Trauma 2007 62 6 Suppl S58 10.1097/TA.0b013e318065ab4e 17556976

[B29] Arozullah AM Daley J Henderson WG Khuri SF Multifactorial risk index for predicting postoperative respiratory failure in men after major noncardiac surgery. The National Veterans Administration Surgical Quality Improvement Program Ann Surg 2000 232 2 242 253 10.1097/00000658-200008000-00015 10903604 PMC1421137

[B30] Arozullah AM Khuri SF Henderson WG Daley J Development and validation of a multifactorial risk index for predicting postoperative pneumonia after major noncardiac surgery Annals of internal medicine 2001 135 10 847 857 11712875 10.7326/0003-4819-135-10-200111200-00005

[B31] Moss M Bucher B Moore FA Moore EE Parsons PE The role of chronic alcohol abuse in the development of acute respiratory distress syndrome in adults Jama 1996 275 1 50 54 10.1001/jama.275.1.50 8531287

[B32] Selzer ML The Michigan alcoholism screening test: the quest for a new diagnostic instrument Am J Psychiatry 1971 127 12 1653 1658 5565851 10.1176/ajp.127.12.1653

[B33] Moss M Guidot DM Steinberg KP Duhon GF Treece P Wolken R Hudson LD Parsons PE Diabetic patients have a decreased incidence of acute respiratory distress syndrome Crit Care Med 2000 28 7 2187 2192 10.1097/00003246-200007000-00001 10921539

[B34] American Thoracic Society/European Respiratory Society International Multidisciplinary Consensus Classification of the Idiopathic Interstitial Pneumonias. This joint statement of the American Thoracic Society (ATS), and the European Respiratory Society (ERS) was adopted by the ATS board of directors, June 2001 and by the ERS Executive Committee, June 2001 Am J Respir Crit Care Med 2002 165 2 277 304 11790668 10.1164/ajrccm.165.2.ats01

